# Reactive Oxygen Species (ROS) Are Not a Key Determinant for Zika Virus-Induced Apoptosis in SH-SY5Y Neuroblastoma Cells

**DOI:** 10.3390/v13112111

**Published:** 2021-10-20

**Authors:** Leila Rodrigues de Mendonça-Vieira, Conceição Elidianne Aníbal-Silva, Armando Menezes-Neto, Elisa de Almeida Neves Azevedo, Nágela Ghabdan Zanluqui, Jean Pierre Schatzmann Peron, Rafael Freitas de Oliveira Franca

**Affiliations:** 1 Oswaldo Cruz Foundation/Fiocruz, Aggeu Magalhães Institute, Recife 50740-465, Brazil; armandomenezes@gmail.com (A.M.-N.); elisaaalmeida@gmail.com (E.d.A.N.A.); 2 Ribeirão Preto Medical School, University of São Paulo, Ribeirão Preto 14049-900, Brazil; elidianneanibal@usp.br; 3 Department of Immunology, Institute of Biomedical Science, University of São Paulo, São Paulo 05508-000, Brazil; nagelaghabdan@gmail.com (N.G.Z.); jeanpierre@usp.br (J.P.S.P.)

**Keywords:** Zika Virus, SH-SY5Y cell line, apoptosis, N-acetylcysteine, oxidative stress

## Abstract

Introduction: ZIKV is a highly neurotropic virus that can cause the death of infected neuroprogenitor cells through mitochondrial damage and intrinsic apoptotic signaling. In this context, the role of reactive oxygen species (ROS) in neuronal cell death caused by ZIKV still remains elusive. Objective: We aimed at evaluating the role of these cellular components in the death of human undifferentiated neuroblastoma cell line infected with ZIKV. Results: ZIKV infection resulted in the extensive death of SH-SY5Y cells with the upregulation of several genes involved in survival and apoptotic responses as well as the colocalization of mitochondrial staining with ZIKV Envelope (E) protein. Notably, levels of intracellular reactive oxygen species (ROS) were not altered during ZIKV infection in undifferentiated SH-SY5Y cells, and consistent with these results, the treatment of infected cells with the widely studied ROS scavenger N-acetylcysteine (NAC) did not prevent cell death in these cells. Conclusion: Altogether, our results suggest that excessive ROS production is not the main trigger of SH-SY5Y cells death in ZIKV infection.

## 1. Introduction

The emergence of Zika Virus (ZIKV) in Latin America was followed by a major economic and social impact, mainly for pregnant women and for the families of children affected by Congenital Zika Syndrome [1]. As a highly neurotropic virus, ZIKV is able to infect the neural progenitor cells of unborn children and interferes with several key cellular processes, such as cell cycle [2], neuronal differentiation [3], and neuronal migration [4]. The loss of these important cellular functions induces premature neuronal death in the forming Central Nervous System (CNS) that underlies congenital neurological manifestations of ZIKV infection [5]. 

Apoptosis is a homeostatic cellular process that occurs during development and aging, but it also occurs as a defense mechanism following sustained proinflammatory responses or cell body damage [6,7]. In this way, programmed cell death plays a central role in controlling viral infection spread, and it can be triggered via two main pathways: (1) the extrinsic pathway, in which an external stress stimulus, e.g., proinflammatory cytokines, triggers the activation of death receptors pathways followed by caspase-8 activation; and (2) the intrinsic pathway, in which a stress signal, e.g., hypoxia-excessive ROS production, triggers mitochondrial membrane damage, releasing cytotoxic mitochondrial components (e.g., cytochrome c) followed by caspase-9 activation [8].

Mitochondrial damage is caused by the loss of membrane integrity due to cell stressors [9]. A previous report showed that the mitochondrial damage and activation of intrinsic apoptotic pathways are triggered by ZIKV infection in neuroprogenitor cells [2]. Another previous study has recently reported that ZIKV infection triggers the intrinsic apoptotic pathway in SH-SY5Y cells with changes in mitochondrial membrane potential, cytochrome c release, and caspase-9 activation, and that proapoptotic protein Bax plays a central role in this process [10]. 

Free radicals usually play an important role as mitochondrial stressors, and the loss of compartmentalization and excessive generation of these radicals can lead to intracellular membrane damage, DNA damage, compromised intracellular reactions, and apoptosis [11]. These features can be observed in cells infected with flaviviruses, as previous reports show that Japanese encephalitis virus (JEV) [12] and Dengue virus 2 (DENV2) infection [13] cause imbalance in host cell free radical homeostasis that is closely related to viral pathogenesis. Similarly, previous reports showed that ZIKV infection induces the upregulation of Mn-superoxide dismutase (Mn-SOD) enzyme expression in monkey kidney cell line LLC-MK2 [14] and mitochondrial damage caused by ROS imbalance in human astrocytes [15]. However, it is still unclear whether ZIKV infection induces excessive ROS generation in neuronal-like cells and its exact impact in cell death.

Considering the multipoint upstream signals that can lead to cell death, our aim was to evaluate the role of reactive oxygen species (ROS) in ZIKV infection of a human undifferentiated neuroblastoma cell line. To this purpose, we used the extensively described human SH-SY5Y cell line as a model to study ZIKV South American strain in vitro infection. Our results suggest that indeed, ZIKV infection can lead to the activation of the intrinsic apoptotic pathway and cell death. Notably, levels of intracellular reactive oxygen species (ROS) were not altered during ZIKV infection in undifferentiated SH-SY5Y cells, and the treatment of infected cells with ROS scavenger NAC had no effect on the cell death levels in these cells. Our results suggest that excessive ROS production is not determinant for the death of SH-SY5Y cells in ZIKV infection.

## 2. Materials and Methods

### 2.1. Cell Culture and Viral Infection

Human neuroblastoma cell line SH-SY5Y (ATCC^®^ CRL-2266™) was grown in Minimum Essential Medium (MEM) (Gibco/Invitrogen, CA, USA) supplemented with Ham’s F12 Nutrient Mixture (Sigma-Aldrich, St. Louis, MO, USA), 10% (*v*/*v*) fetal bovine serum (FBS), 0.1 mM MEM non-essential amino acids (Gibco/Invitrogen, CA, USA), 1 mM sodium pyruvate (Sigma-Aldrich, St. Louis, MO, USA), 2 mM L-glutamine (Gibco/Invitrogen, CA, USA), 100 U/mL penicillin (Gibco/Invitrogen, CA, USA), and 0.1 g/mL streptomycin (Gibco/Invitrogen, CA, USA), at 37 °C with 5% CO_2_ environment. We optimized SH-SY5Y cell culture protocol in order to minimize the number of passages in our in vitro cell culture system, and cell morphology was closely analyzed in each passage to verify the presence of its neuroblast-like characteristics morphological features [16]. For all experiments, cells were grown for 24–30 h in culture conditions before each experiment, and the maximum number of passages of cells employed in all experiments was 20. 

The ZIKV PE243 strain was originally isolated in C6/36 cells from a patient with symptoms of febrile illness as previously reported [17] and was further propagated by conducting four passages in VERO cells. Viral stocks were clarified by centrifugation (2000× *g* at 4 °C for 10 min), filtered in Millipore Express^®^ PES Membrane Filters (0.22 µm) (Millipore, CO, USA), and stored at −80 °C. Virus titer was determined by plaque assay in VERO cells.

SH-SY5Y monolayers were infected with ZIKV stocks at different Multiplicities of Infection (MOI), in a small volume of culture medium containing 2% (*v*/*v*) FBS, for 1–2 h at 37 °C. After virus adsorption, cells were replenished with fresh medium containing 2% (*v*/*v*) FBS and incubated at 37 °C with 5% CO_2_. At the indicated time points, ZIKV- and mock-infected cells (medium without virus) were harvested and processed for further analysis.

### 2.2. ZIKV Titration in SH-SY5Y Cells by Plaque Assay

Mock- and ZIKV-infected (MOI 0.5) SH-SY5Y cell supernatant samples were harvested at 2 dpi. Confluent monolayers of VERO cells were inoculated with 10-fold serial dilution of supernatant samples from SH-SY5Y supernatant samples (each dilution point was performed in duplicate) and incubated for 2 h at cell culture conditions. After incubation, inoculums were removed, and cells were replenished with Minimum Essential Medium (MEM) containing 2.5% carboxymethyl cellulose (CMC) and 2% FBS. After an incubation period of 6 days, cell monolayers were fixed using 10% (*v*/*v*) formalin diluted in phosphate-buffered saline (PBS), stained with crystal violet solution, and plaque numbers were counted. Viral titer was calculated in PFU mL^−1^ using the following formula: T = P/(D × V), where P is the average number of plaques in the duplicate, D is the dilution factor, and V is the volume of diluted virus added per well.

### 2.3. ZIKV Replication Kinetics by Real-Time PCR

Supernatants from mock- and ZIKV-infected samples were harvested at the indicated timepoints and stored at −80 °C. Viral RNA was extracted using a QIAamp Viral RNA Mini kit (QIAGEN, Hilden, Germany). Complementary DNA (cDNA) was synthesized with a High-Capacity cDNA Reverse Transcription Kit (Thermo Fisher Scientific, Waltham, MA, USA). A ZIKV stock with a known viral titer was used to prepare a relative standard curve for estimation of the number of viral genome copies in each reaction. A standard curve was prepared by performing 10-fold serial dilutions of the viral stock RNA sample, which was followed by relating original viral stock titer in PFU with the qPCR cycle threshold (CT) amplification of each point of the dilution curve. Quantitative RT-PCR (RT-qPCR) reactions were performed with the QuantiNovaTM Probe RT-PCR Kit (QIAGEN, Hilden, Germany), ZIKV-specific primers and probes were employed, as previously described [18]. Reactions were performed with the ABI 7500 Real-Time PCR System (Thermo Fisher Scientific, Waltham, MA, USA) under the following cycling conditions: 45 °C for 10 min; 95 °C for 5 min; 95 °C for 5 s; and 60 °C for 45 s (45 cycles).

### 2.4. Analysis of Cell Death Markers by Flow Cytometry (FCM) 

Mock- and ZIKV-infected (MOI 5) SH-SY5Y cells (6 × 10^4^) grown in 48-well plates were harvested at the indicated times post-infection, washed with PBS (phosphate buffered saline), and processed for FCM analysis. For cell death quantification, cells were stained with Annexin V (FITC) and 7-Aminoactinomycin D (7-AAD), according to the manufacturer’s instructions (BD Pharmingen, San Jose, CA, USA). For caspase-3/7 and SYTOX staining, cells were processed using a CellEvent™ Caspase-3/7 Green Flow Cytometry Assay Kit (Molecular Probes, Invitrogen, OR, USA) according to the manufacturer’s instructions. For cleaved PARP labeling, cells were labeled with mouse anti-human cleaved PARP (1:20, #552933, BD Biosciences, Franklin Lakes, NJ, USA) and 7-AAD, according to the manufacturer’s instructions. All FCM analysis was performed using BD FACSAriaIII Cell Sorter (BD Biosciences, Franklin Lakes, NJ, USA) and data analysis was performed using BD FACSDiva (BD Biosciences, Franklin Lakes, NJ, USA) and FlowJo (TreeStar, Woodburn, OR, USA) software. 

### 2.5. Intracellular Cytokine Staining and FCM Analysis

Mock- and ZIKV-infected (MOI 5) SH-SY5Y cells were harvested at the indicated timepoints, incubated at 37 °C with BD GolgiPlug™ Protein Transport Inhibitor containing Brefeldin A (BD Biosciences, Franklin Lakes, NJ, USA) for 1 h prior to fixation. This step enables the accumulation of cytokines in the Golgi complex, facilitating the detection of cytokine-producing cells. Then, cells were fixed and permeabilized using BD Cytofix/Cytoperm™ Fixation/Permeabilization Solution Kit (BD Biosciences, Franklin Lakes, NJ, USA), following the manufacturer’s instructions. The following antibodies were diluted 1:20 in BD Perm/Wash™ (BD Biosciences, Franklin Lakes, NJ, USA) and used for labeling: PE-conjugated anti-FasL (CD178, BioLegend #106606); APC-conjugated anti-TRAIL (CD253); PE-conjugated anti-TNF-α (BioLegend #506306); PE-conjugated anti-cleaved PARP (BD Biosciences #552933). Samples were incubated with antibody solution for 10–15 min and then washed with PBS. FCM analysis was performed using the BD FACSAriaIII Cell Sorter (BD Biosciences, Franklin Lakes, NJ, USA). Results were analyzed using BD FACSDiva (BD Biosciences, Franklin Lakes, NJ, USA) and FlowJo (TreeStar, Woodburn, OR, USA) software.

### 2.6. ZIKV E Protein Labeling and FCM Analysis

Mock- and ZIKV-infected (MOI 5) SH-SY5Y cells were harvested at the indicated timepoints, cells were fixed using a BD Cytofix/Cytoperm™ Plus Fixation/Permeabilization Kit, and intracellular antibody labeling was performed following the manufacturer’s instructions (BD Biosciences, Franklin Lakes, NJ, USA). Cells were labeled with the 4G2 mouse anti-flavivirus E monoclonal primary antibody and goat anti-mouse IgG FITC-conjugated secondary antibody (Sigma-Aldrich #F0257, 1:100). Samples were incubated with antibody solutions for 30 min and then washed with PBS. FCM analysis was performed using the BD FACSAriaIII Cell Sorter (BD Biosciences, Franklin Lakes, NJ, USA). Results were analyzed using BD FACSDiva (BD Biosciences, Franklin Lakes, NJ, USA) and FlowJo (TreeStar, Woodburn, OR, USA) software.

### 2.7. Immunofluorescence Reaction and Confocal and Epifluorescence Microscopy Assays

Mock- and ZIKV-infected (MOI 5) SH-SY5Y cells (1 × 10^5^) grown in 0.13 mm round glass slides were processed at 2 dpi for mitochondrial staining using a Mitotracker™ Deep Red FM probe (Molecular Probes, Invitrogen, OR, USA). Briefly, cells were incubated with 500 µM of mitotracker probe for 45 min and then fixed in PBS/3% formaldehyde for 15 min at room temperature (RT). For antibody labeling, cells were fixed in PBS/4% paraformaldehyde for 15 min at RT and then permeabilized with PBS/0.2% Triton X-100 (Sigma-Aldrich, St. Louis, MO, USA) for 10 min at RT. Slides containing the fixed cells were blocked with PBS/porcine gelatin solution (0.2%) (Sigma-Aldrich, St. Louis, MO, USA) for 30 min at 37 °C. After blocking step, cells were incubated with primary antibody for 1 h at 37 °C as follows: mouse anti-flavivirus E protein monoclonal antibody 4G2 (1:10, ATCC: HB-112). Then, cells were incubated with AlexaFluor 488-conjugated donkey anti-mouse IgG secondary antibody (1:1000, Molecular Probes, Invitrogen, OR, EUA) for 1 h at 37 °C. Nuclei were stained with Hoechst 33342. Samples were mounted using ProLong^®^ Diamond Antifade Mountant (Molecular Probes, Eugene, OR, USA) or Fluoromount G mounting medium (EM Sciences, PA, USA) and analyzed using Leica DMI4000 microscope or SPII2-AOBS confocal laser scanning microscope (Leica Microsystems, Wetzlar, Germany). Images were processed and analyzed using ImageJ (National Institutes of Health, Bethesda, MD, USA).

### 2.8. Pharmacological Inhibition Assays 

For caspase inhibition treatment, mock- and ZIKV-infected (MOI 1 and 5) SH-SY5Y cells were treated with Q-VD-OPh (Sigma-Aldrich, St. Louis, MO, USA) at 10 μM or 25 μM (as indicated in figure legends). Q-VD-OPh stock solution was prepared in DMSO. Control samples were media-treated or vehicle-treated (DMSO final concentration ≤ 0.01%), and the cell media was replenished every two days. At indicated timepoints, cells were harvested and stained with Annexin-V-FITC and 7-AAD and analyzed by FCM, or cells were harvested and immediately processed using CellTiter 96^®^ Aqueous One Solution Cell Proliferation Assay according to the manufacturer’s instructions (Promega, Madison, WI, USA). Absorbance values at 490 nm of all experimental conditions were normalized according to the following parameters: absorbance values of ZIKV-infected untreated samples were set to 0%; and absorbance values of mock-infected untreated samples were set to 100%. 

For ROS scavenging assay, NAC (Sigma-Aldrich, St. Louis, MO, USA) was diluted in sterile water or cell culture medium at concentrations of 500 μM, 750 μM, and 1 mM. ZIKV-infected cells were treated 2 h before or immediately after viral inoculation. After 2 h of viral inoculation, cell culture media only (mock-treated) or containing NAC (NAC-treated) was renewed in both mock- and ZIKV-infected samples, and samples were incubated for the indicated times post-infection. Treated and mock-treated cell culture media were renewed every two days, and cells were stained with Annexin V-FITC and 7-AAD after the indicated timepoints and analyzed by FCM. 

### 2.9. SDS-PAGE and Immunoblot 

SH-SY5Y cells (1 × 10^6^) were grown in 25 cm^2^ cell culture flasks and infected with ZIKV (MOI 0.5). Cell pellets were harvested at indicated timepoints and lysed in RIPA buffer (Cell Signaling, Danvers, MA, USA) containing a protease inhibitor cocktail (GE Healthcare Life Sciences, Chicago, IL, USA) for 20 min at 4 °C. Samples were further centrifuged at 14,000 rpm for 20 min at 4 °C, supernatants were harvested, and protein concentration was determined using Bradford reagent (Sigma-Aldrich, St. Louis, MO, USA), according to the manufacturer’s instructions. Samples were heated at 90 °C for 5 min, 10 μg of total protein was loaded in each lane, and protein separation was performed by SDS-PAGE on 4–15% polyacrylamide gels (Bio-Rad Laboratories, Hercules, CA, USA). Proteins were transferred to 0.45 μm PVDF membranes and efficiency was assessed by 0.5% Ponceau S (Sigma-Aldrich, St. Louis, MO, USA) in 5% acetic acid. Membranes were stored dry at 4 °C for at least 16 h before immunostaining. Before labeling, membranes were washed with 0.1% Tween 20 (Sigma-Aldrich, St. Louis, MO, USA) in PBS (PBS-Tween) and incubated in blocking solution (1%, 5% or 7.5% skimmed milk powder diluted in PBS-Tween) for 2–8 h and then washed five times for 5 min with PBS-Tween. Labeling was performed using the following antibodies and dilutions: anti-human APAF1 (1:1000, 5088S, Cell Signaling, Danvers, MA, USA), anti-human procaspase-9 and cleaved caspase-9 (1:1000, 9508S, Cell Signaling, Danvers, MA, USA), and anti-human GAPDH (1:3000–1:5000, 9545, Sigma-Aldrich, St. Louis, MO, USA). After 12–16 h of incubation with primary antibodies at 4 °C, membranes were washed with PBS-Tween and incubated with appropriate HRP-conjugated secondary antibody diluted in PBS-Tween containing 7.5% skimmed milk powder for 1 h at RT. After thorough washing in PBS-Tween, antibody labeling was detected by autoradiography film (GE Healthcare Life Sciences, Chicago, IL, USA) or a ChemiDoc™ XRS+ system (Bio-Rad Laboratories, Hercules, CA, USA), using ECL solution (GE Healthcare Life Sciences, Chicago, IL, USA). Densitometry analysis was performed using ImageJ (National Institutes of Health, Bethesda, MD, USA) and ImageLab (Bio-Rad Laboratories, Inc., Hercules, CA, USA) software. Protein fold-change was calculated as follows: (1) protein expression values were calculated by dividing the intensity values for a target protein by the intensity values of the internal loading control protein (i.e., GAPDH) for each sample; and (2) fold-change was calculated by dividing the expression values of ZIKV-infected samples at each timepoint post-infection by the average of the expression values of mock-infected samples.

### 2.10. Gene Expression Analysis by qRT-PCR

SH-SY5Y cells (1 × 10^6^ or 2.5 × 10^6^) were grown in 25 cm^2^ cell culture flasks and infected with ZIKV (MOI 0.5). Mock- and ZIKV-infected cells were harvested at the indicated timepoints and processed for total RNA extraction using RNeasy Micro Kit (QIAGEN, Hilden, Germany). Complementary DNA (cDNA) synthesis was performed using a High-Capacity cDNA Reverse Transcription Kit (Thermo Fisher Scientific, Waltham, MA, USA), according to the manufacturer’s instructions. The quality of cDNA samples was evaluated using a Thermo Scientific™ NanoDrop™ spectrophotometer (Thermo Fisher Scientific, Waltham, MA, USA), analyzing absorbance values at 260 nm/280 nm and 260 nm/230 nm. cDNA samples were quantified using the Qubit™ dsDNA HS Assay Kit (Thermo Fisher Scientific, Waltham, MA, USA) and analyzed with a Qubit fluorometer (Thermo Fisher Scientific, Waltham, MA, USA). 

Gene expression analysis was performed using TaqMan ™ Array Human Apoptosis via Death Receptors (Thermo Fisher Scientific, Waltham, MA, USA), which consists of 96-well plates pre-configured with 42 sets of probes and primers specific to genes involved in apoptosis and survival response pathways (Supplemental Table S1) and six endogenous control candidate genes (*ACTA1*, *ACTB*, *18S*, *GAPDH*, *GUSB*, *HPRT*). The amount of cDNA samples added per reaction was 3.5 ng and qPCR reactions were performed using an ABI 7500 Real-Time PCR System (Thermo Fisher Scientific, Waltham, MA, USA). Gene expression analysis was performed using 7500 v2.0.6 (Thermo Fisher Scientific, Waltham, MA, USA), Microsoft Excel, and GraphPad Prism (Graph Pad Software Inc., San Diego, CA, USA) software. 

Among endogenous control candidates, *GUSB* showed the most stable expression in our experimental conditions (standard deviation = 0.26) and was thus selected as the endogenous control gene. Analysis of gene expression levels was performed as follows: (1) Amplification CT values ≤ 36 were considered as above detection limit, and CT values ≥ 36 were considered below the detection limit and set to 36 for the purpose of gene expression analysis; (2) CT values of target genes were subtracted from the CT values of the endogenous control gene, resulting in ΔCT values; (3) ΔCT values from infected samples were subtracted from ΔCT values from uninfected control samples, resulting in ΔΔCT values; (4) 2[-ΔΔCT] values for each condition were considered as gene expression fold-change. Gene expression data are deposited on the Gene Expression Omnibus online repository under the accession number GSE162096.

### 2.11. Reactive Oxygen Species (ROS) Detection Assay

Mock- and ZIKV-infected (MOI 1, 10, or 100) SH-SY5Y cells were harvested at the indicated timepoints, and ROS detection was performed using DCFDA Cellular ROS Detection Assay Kit (Abcam, Cambridge, UK), following the manufacturer’s instructions. Briefly, cells were incubated with DCFDA for 45 min, washed with PBS, and FCM analysis was performed using Accuri C6 cytometer (BD Biosciences, Franklin Lakes, NJ, USA). Data analysis was performed using BD FACSDiva (BD Biosciences, Franklin Lakes, NJ, USA) and FlowJo (TreeStar, Woodburn, OR, USA) software.

### 2.12. Statistical Analysis

Data are shown as mean ± standard deviation (SD). Shapiro–Wilk and Kolmogorov–Smirnov normality tests were performed before statistical analysis with parametric tests. Statistical analysis was performed using Student’s t-test or variance analysis (one-way or two-way ANOVA) followed by Tukey’s multiple comparisons test, as indicated in the figure legends. All statistical analyses were performed using GraphPad Prism 8 software (GraphPad Software, Inc., La Jolla, CA, USA), and the results were considered significant when the *p*-value was below 0.05 (significance level of 5%).

## 3. Results

### 3.1. ZIKV-Induced Apoptosis Is Partially Dependent on Caspase Activation in SH-SY5Y Cells

**Figure 1:**
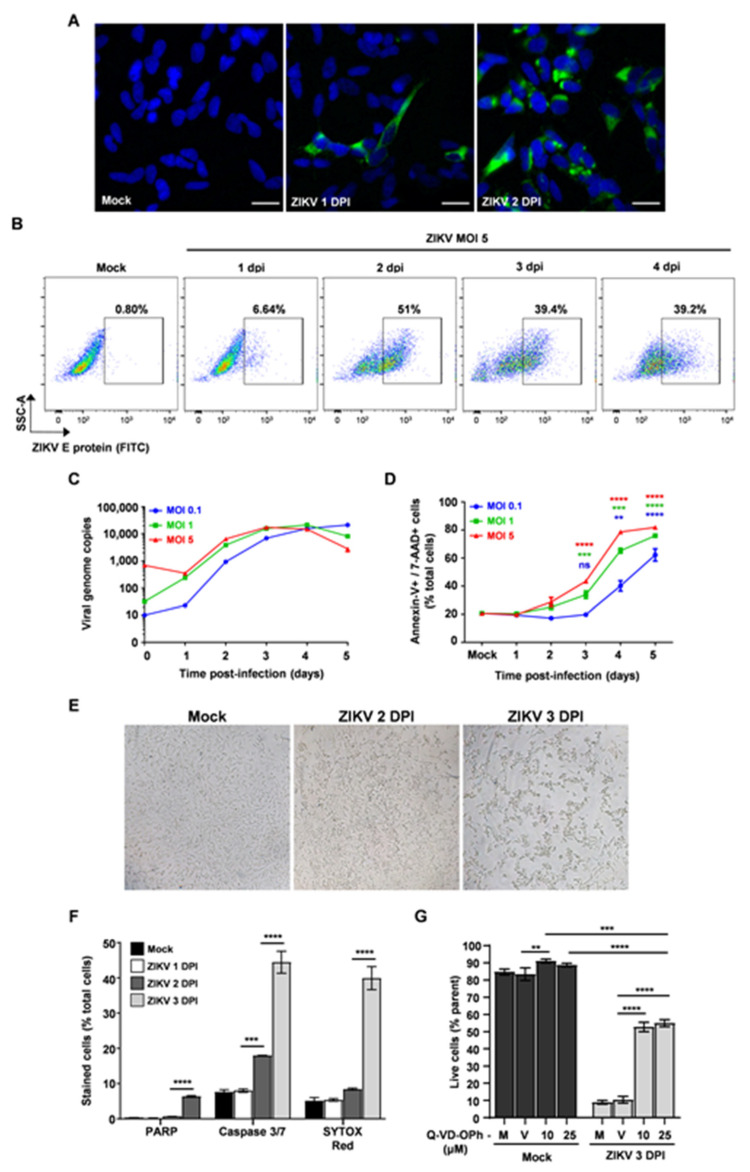
ZIKV infection in SH-SY5Y cells induces apoptosis partially dependent on caspase activation. (**A**) Immunofluorescence of mock- and ZIKV-infected (MOI 5) cells analyzed at 2 dpi. Antibody labeling was performed with anti-flavivirus E (4G2) primary antibody and AlexaFluor-488 anti-mouse IgG secondary antibody. Nuclei were stained with Hoechst 33342 (blue). Scale bar 20 μM. Representative data of three independent experiments performed in duplicates. (**B**) FCM analysis of mock- and ZIKV-infected (MOI 5) cells labeled with anti-flavivirus E (4G2) primary antibody and anti-mouse IgG FITC-conjugated secondary antibody. (**C**) ZIKV growth curve at MOI 0.1 (blue), 1 (green), and 5 (red), assessed by qRT-PCR in cell supernatant. Data represented as mean ± SD of two independent experiments performed in triplicate. (**D**) Annexin-V+/7-AAD+ double-positive cells frequency (percentage of total cells). Data are represented as mean ± SD of two independent experiments performed in triplicate. Statistical analysis performed by one-way ANOVA followed by Tukey’s post-test. ** *p* < 0.01, *** *p* < 0.001, **** *p* < 0.0001, ns—not-significant, compared to mock-infected, according to Tukey’s post-test. (**E**) Light microscopy of mock- and ZIKV-infected SH-SY5Y cells (MOI 5) at 3 dpi. Magnification 80X. Inset magnification: 160X. dpi—days post-infection. (**F**) Mock- and ZIKV-infected (MOI 5) SH-SY5Y cells were labeled with anti-cleaved PARP PE-conjugated antibody or stained with four-amino acid peptide (DEVD) conjugated to a nucleic acid-binding dye and SYTOX™ AADvanced™ Dead Cell Stain. Then, cells were analyzed by FCM at the indicated timepoints. Values represent frequency of positive cells within the total cell population of mock- and ZIKV-infected cells. Data represented as mean ± SD of two independent experiments performed in triplicate. Statistical analysis performed by one-way ANOVA followed by Tukey’s post-test. *** *p* < 0.001, **** *p* < 0.0001, according to Tukey’s post-test. (**G**) Mock- and ZIKV-infected (MOI 5) SH-SY5Y cells were treated with Q-VD-OPh pan caspase inhibitor (10 μM and 25 μM), and cell death was assessed by annexin-V/7-AAD staining and FCM analysis at 3 dpi. The graph shows the frequency of double-positive (annexin-V+ and 7-AAD+) cells within the total cell population of mock- and ZIKV-infected cells (%parent). M—media-treated cells; V—vehicle-treated cells (DMSO); DPI—days post-infection. Data represented as mean ± SD. ** *p* < 0.01 and **** *p* <0.0001, according to one-way ANOVA followed by Tukey’s post-test. Averaged data of three independent experiments performed in triplicate or quadruplicate.

As reported previously, ZIKV efficiently infects human and mouse neuronal progenitor cells leading to cell dysfunction, apoptosis, and inhibition of neuronal differentiation [19,20,21]. Here, we used a human undifferentiated neuroblastoma cell line denominated SH-SY5Y to model ZIKV infection. First, we analyzed the virus growth in SH-SY5Y cells by infecting these cells with a high viral load (MOI 5) and detected an increase in the number of envelope (E) protein-positive cells over time, mainly between 1 and 2 dpi (Figure 1A,B). Next, we infected cells with low (MOI 0.1) to high (MOI 5) viral loads and detected a significant increase in viral RNA levels in cell supernatant between 1 and 2 dpi, reaching a growth plateau at 3 and 4 dpi (Figure 1C). ZIKV infection at MOI 0.5 leads to the production of infectious particles, although it was not highly productive (less than 2 × 10^4^ at 2 dpi) (Supplemental Figure S1A). Infected cells showed a significantly higher death rate as evidenced by Annexin-V/7-AAD staining at 3 and 4 dpi (Figure 1D) and by morphological changes suggestive of apoptotic cells mainly at 3 dpi (Figure 1E). The extensive cell death rate observed at these timepoints is associated with a decline in ZIKV growth and in the rate of ZIKV-infected cells, as observed in Figure 1B,C. Next, to better characterize the intracellular processes engaged in ZIKV-induced cell death, we assessed the downstream signaling pathways that could be activated during infection. We observed a significant increase in the fluorescence levels of cleaved nuclear protein poly-(ADP-ribose)-polymerase (PARP) and SYTOX, which is a high-affinity nucleic acid stain that penetrates cells with compromised membrane integrity, mainly between 2 and 3 dpi (Figure 1F). This process was preceded by a significant increase of activated caspase-3/7 observed as early as 2 dpi (Figure 1F). In addition, the blockage of caspases activation with a pan-caspase inhibitor (Q-VD-OPh) significantly increased the survival rate of infected cells by nearly 50% at 3 dpi compared to media-treated cells (M) or vehicle-treated cells (V) (Figure 1G, and Supplemental Figure S1B), and this protective effect was not related to a diminished virus replication rate (Supplemental Figure S1C). Thus, these results indicate that caspase-3/7 activation plays an important, albeit not exclusive, role in triggering cell death, and that PARP cleavage occurs subsequently to caspase-3/7 activation as a downstream process of cell death.

### 3.2. ZIKV Infection Triggers Intrinsic Apoptotic Pathway

**Figure 2:**
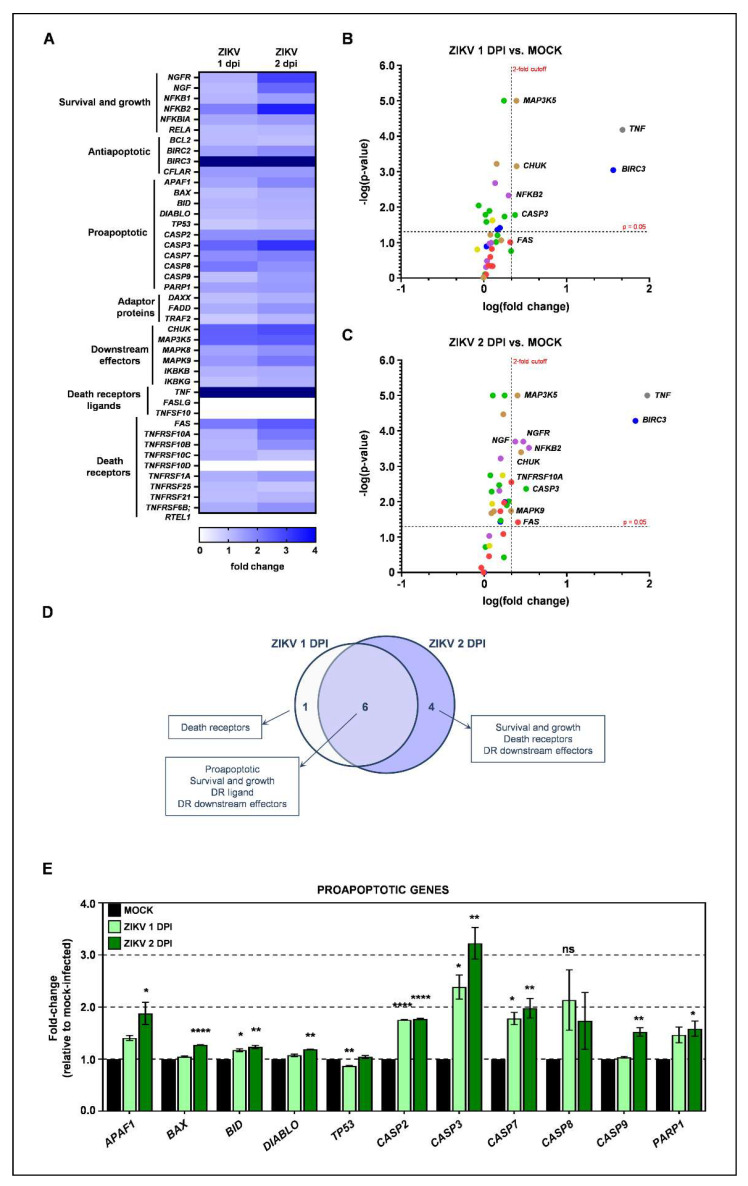
ZIKV infection modulates the expression of genes related to cell survival and cell death pathways. Mock- and ZIKV-infected (MOI 5) SH-SY5Y cells were harvested and processed at 1 and 2 dpi for gene expression analysis by qPCR. (**A**) Fold-change heatmap of target genes. Color coding: white: gene expression not detected; dark blue: genes with fold-change of more than four-fold. (**B**,**C**) Volcano plots of fold-change with respective statistical significance analyzed at 1 dpi (**B**) and 2 dpi (**C**), according to unpaired t-test compared to mock-infected samples. Color coding: (**D**) Venn diagram showing numbers of genes with expression variation of more than two-fold at each or both of the analyzed timepoints and information on gene function. (**E**) Expression levels of proapoptotic genes at 1 (light green bars) and 2 dpi (dark green bars). Data represented as mean ± (SD) of one experiment performed in duplicate. Statistical analysis performed by one-way ANOVA followed by Tukey’s post-test. ns—non-significant, * *p* < 0.05, ** *p* < 0.01, **** *p* < 0.0001, according to Tukey’s post-test.

We next analyzed the expression profile of genes related to survival and death signaling pathways during ZIKV infection in SH-SY5Y cells. Considering that extensive cell death is already observed at 3 dpi (Figure 1D,E), we sought to assess the gene expression profile before cell death onset to better encompass the primary signaling pathways triggered early in ZIKV infection. Hence, gene expression profiling was performed at 1 and 2 dpi in cells infected with MOI 5, since cell death and SYTOX Red staining are almost at baseline levels at these timepoints (Figure 1D,F,G). This analysis included a total of 42 genes related to the death receptors’ signaling pathway and survival as well as anti-apoptotic and proapoptotic responses (Figure 2, Supplemental Figure S2 and Supplemental Table S1). Although the cell death process is not apparent at the analyzed timepoints (Figure 1D–F), a total of 32 genes was already significantly modulated at either 1 or 2 dpi, among which 13 genes were modulated at 1 dpi (12 genes upregulated and one gene downregulated) and 29 genes upregulated at 2 dpi (Figure 2A,B,C,E, Supplemental Figure S2 and Supplemental Table S1). Among genes upregulated by ZIKV infection, only 11 genes showed significant upregulation equal or greater than two-fold at either 1 and/ or 2 dpi (Figure 2B–D).

**Figure 3:**
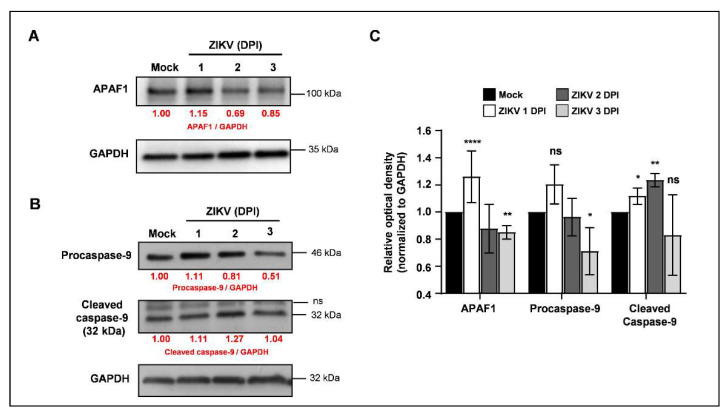
ZIKV infection triggers intrinsic apoptotic pathway. Mock- and ZIKV-infected (MOI 5) SH-SY5Y cells were harvested at the indicated timepoints and processed for immunoblotting analysis. Membranes were labeled with (**A**) anti-APAF1, (**B**) anti-caspase-9, and anti-GAPDH primary antibodies and appropriate HRP-conjugated secondary antibodies. Values in red show protein fold-change relative to mock-infected samples. Densitometry values were normalized to GAPDH before expression analysis. kDa—kilodaltons. (**C**) Relative quantification of densitometry analysis. Values are represented as mean ± S.D. Data representative of three independent experiments performed in triplicate. * *p* < 0.05, ** *p* < 0.01, **** *p* < 0.0001, ns—not significant, according to unpaired t-test compared to mock-infected.

Among genes involved in survival and antiapoptotic responses, *BIRC3* showed strong upregulation at 1 and 2 dpi, whereas *BIRC2*, *CFLAR*, *NFKB1,* and *NFKBIA* showed significant upregulation of less than two-fold and *NFKB2*, *NGF,* and *NGFR* showed upregulation greater than two-fold at 2 dpi (Supplemental Figure S2A,B). Among proapoptotic genes, *CASP3* was the only gene with two-fold upregulation starting at 1 dpi and increasing at 2 dpi (Figure 2E), which also coincides with caspase-3/7 activation (Figure 1G). *APAF1*, *BAX*, *BID*, *DIABLO*, *CASP2*, *CASP3*, *CASP9,* and *PARP1* were also significantly upregulated during infection, and *TP53* was the only proapoptotic gene slightly downregulated at 1 dpi with unremarkable modulation at 2 dpi (Figure 2E). Although upregulation of the *APAF1* gene was detected at 2 dpi, a significant increase in APAF1 protein levels was already detectable at 1 dpi (Figure 3A,C), together with a significant increase in cleaved caspase-9 levels at 1 and 2 dpi followed by a significant decrease in procaspase-9 levels at 3 dpi (Figure 3B,C). This is also followed by a notable intracellular redistribution of mitochondrial staining that was also observed, showing a clustered staining pattern juxtaposed to the nucleus of ZIKV-infected cells at 2 dpi (Supplemental Figure S3, lower panels) in contrast to the widespread filament-like staining pattern observed in uninfected cells (Supplemental Figure S3, upper panels). These results indicate that ZIKV infection in SH-SY5Y cells triggers caspase-9 activation and an intrinsic apoptotic pathway.

Among the genes involved in the death receptors pathway with modulation of more than two-fold, *CHUK*, *MAP3K5*, and *TNF* were upregulated at both 1 and 2 dpi, and *FAS*, *MAPK9,* and *TNFRSF10A* were upregulated only at 2 dpi (Supplemental Figure S2C–E). The expression of *TNFRSF10D*, *TNFSF10* (TRAIL/APO2L encoding gene), and *FASLG* (FasL/CD95L encoding gene) were not detected in either mock- or ZIKV-infected cells (Supplemental Figure S2C,D). Seven other genes involved in the death receptors pathway were also significantly upregulated albeit less than two-fold (Supplemental Figure S2C,E,F). Then, we analyzed intracellular levels of TNF-α protein and three other death receptors ligands, FasL/CD95L, TWEAK/ APO3L, and TRAIL/APO2L. TRAIL/APO2L levels were unaltered at 1 dpi and FasL/CD95L, TWEAK/ APO3L, and TNF-α levels remained unaltered throughout the first 48 h of ZIKV infection (Supplemental Figure S4A–D). Moreover, despite the strong upregulation of TNF-α encoding gene at 1 and 2 dpi (Supplemental Figure S2D), soluble TNF-α was not detected in the supernatant of both mock- and ZIKV-infected cells at any of the analyzed timepoints post-infection (Supplemental Table S2). To further assess the possible role of TNF-α in ZIKV-induced SH-SY5Y cell death, we blocked TNF-α synthesis by treating mock- and ZIKV-infected cells with pentoxifylline (PTX), which is a widely used phosphodiesterase inhibitor that inhibits TNF-α synthesis possibly at the transcriptional level [22,23]. PTX treatment did not prevent death caused by ZIKV infection in SH-SY5Y cells, as quantified by LIVE/DEAD staining followed by FCM analysis (Supplemental Figure S4E,F), suggesting that TNF-α synthesis does not seem to be determinant for ZIKV-induced apoptosis in SH-SY5Y cells.

### 3.3. NAC Treatment Is Not Able to Reverse Cell Death in SH-SY5Y Cells Infected with ZIKV

**Figure 4:**
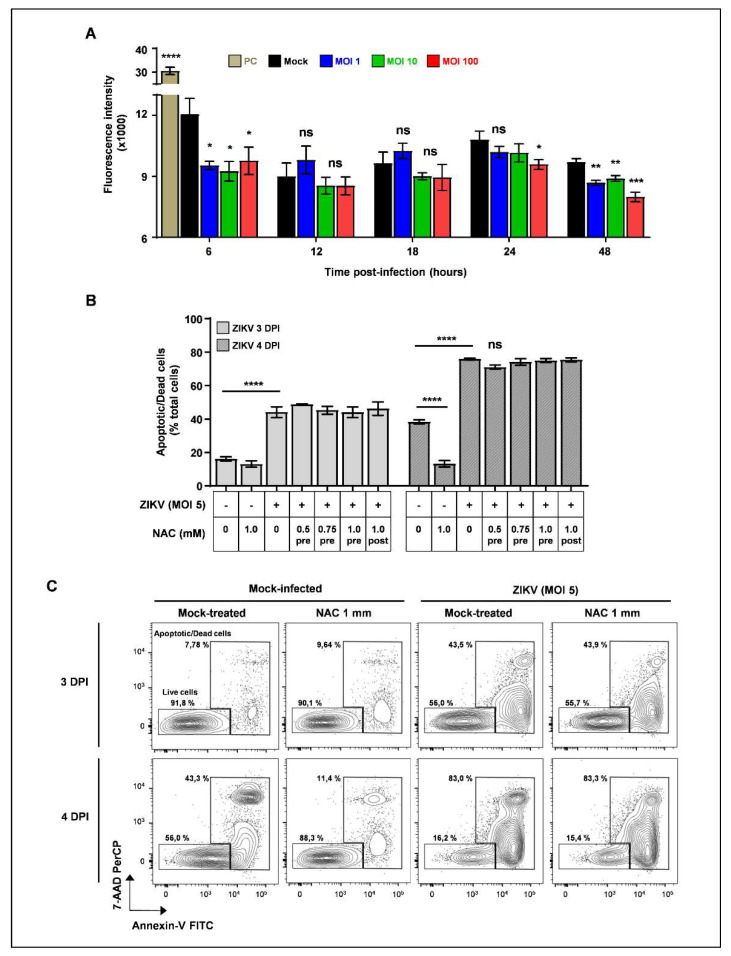
ZIKV-induced cell death is independent of ROS production. (**A**) Mock- or ZIKV-infected (MOI 1, 10, and 100) SH-SY5Y cells were harvested at the indicated timepoints post-infection and stained with DCFDA. PC—positive control (tbHP, tert-butyl hydroperoxide). Values are represented as mean ± S.D. Data representative of one experiment are performed in triplicate. ns—non-significant, * *p* < 0.05, ** *p* < 0.01, *** *p* < 0.0001, according to unpaired t-test compared to mock-infected. **** *p* < 0.0001, according to one-way ANOVA followed by Tukey’s post-test compared to both mock- and ZIKV-infected samples. (**B**,**C**) Mock- and ZIKV-infected (MOI 5) SH-SY5Y cells treated with NAC (0.5 mM, 0.75 mM, and 1 mM) were harvested at the indicated timepoints post-infection, stained with Annexin-VI/7-AAD, and analyzed by FCM. (**B**) Frequency of double-positive cells (Annexin-V+/7-AAD+) within total cell population. Data are represented as mean ± SD of one experiment performed in triplicate. Pre—treatment before viral inoculation; post—treatment after viral inoculation. (**C**) Representative dot plots showing frequency of live (double-negative Annexin-V-/7-AAD-) and dead (double-positive Annexin-V+/7-AAD+) cells within mock- or NAC-treated (1 mM) samples stained with annexin-V/7-AAD and analyzed by FCM.

Considering that a common trigger of intrinsic apoptotic pathway is excessive free radical generation [24,25], we quantified ROS intracellular levels throughout the first 48 h of infection in ZIKV-infected SH-SY5Y cells. ROS levels were significantly decreased mainly at 48 hpi, and this decrease was notably observed in cells infected with higher MOI (Figure 4A). To further assess whether ROS production would be associated with cell death in ZIKV infection, we treated mock- and ZIKV-infected cells with the antioxidant ROS scavenger NAC, and remarkably, NAC treatment did not affect cell death caused by ZIKV infection at any of the tested concentrations and analyzed timepoints post-infection, (Figure 4B,C, and Supplemental Figure S5A). Moreover, the cell death kinetics of ZIKV-infected NAC-treated samples is comparable to the cell death kinetics of ZIKV-infected mock-treated samples at any of the analyzed NAC concentrations (Supplemental Figure S5B). These results suggest that excessive ROS is probably not the main trigger of the intrinsic apoptotic pathway in SH-SY5Y cells infected with ZIKV.

## 4. Discussion

Herein, we demonstrate that a contemporary South American strain of ZIKV is able to infect human undifferentiated SH-SY5Y cells, resulting in extensive cell death, mainly through the engagement of intrinsic apoptosis, further corroborating previous evidence [10]. Previous reports showed that ZIKV-induced neuronal cell death is largely dependent on caspases activation, mainly caspase-3/7 and -9, and it also involves PARP cleavage [26,27,28,29]. Our findings support these findings and also indicate that caspase-3/7 activation is an upstream step to PARP cleavage in ZIKV infection, considering that the blockage of caspase activation only partially rescued cells from death. These data suggest that ZIKV infection can also possibly result in a non-canonical (caspase independent) cell death mechanism.

Mitochondria seem to play a central role in ZIKV-induced cell death in SH-SY5Y cell line, as the redistribution of mitochondrial staining intracellular distribution is altered in ZIKV-infected cells. Previous reports showed that ZIKV and DENV nonstructural protein NS4B disturbs mitochondrial morphodynamics [30]. Changes in the mitochondrial membrane potential directly affect mitochondrial function, causing disturbances in the mitochondrial homeostasis and leading to mitochondrial cytochrome c release [31]. This is also a common feature of other flaviviruses infections. JEV, DENV2, and West Nile virus (WNV) infections induce caspase-9 activation and cytochrome c release in human medulloblastoma cells [32,33,34]. Thus, we suggest that the apoptosis of human neuroblastoma SH-SY5Y cells infected with ZIKV is mainly caused by the disruption of mitochondrial homeostasis and activation of the intrinsic apoptotic pathway.

Here, we found that TNF transcript levels were upregulated after ZIKV infection, although we did not detect significant intracellular and soluble levels of TNF-α protein, and treatment with TNF-α synthesis inhibitor PTX did not affect ZIKV apoptosis in SH-SY5Y cells. TNF-α is synthesized as an integral membrane protein, which is further cleaved in its soluble form by TNF-α converting enzyme (TACE), which is a plasma membrane member of the ADAM family of metalloproteases [35]. Considering this, our theory is that post-transcriptional regulation and silencing mechanisms may be playing a role in regulating levels of TNF-α [36,37,38,39], and further studies are needed in order to investigate this possibility. Furthermore, our results suggesting that TNF-α is not a determinant for cell death triggered in ZIKV infection are in consonance with two previous studies reporting that the inhibition of caspase-8 activation did not prevent cell death during ZIKV infection, further suggesting that the extrinsic (death receptors) apoptotic pathway is not triggered in these experimental conditions [2,10]. Moreover, a previous study reported a lack of FasL and TNF-α production during ZIKV infection in human neural progenitor cells [40], and another previous report showed that a model of primary human astrocytes also failed to produce detectable levels of TNF-α following ZIKV infection [41], further corroborating our data. Conversely, a previous report showed that ZIKV infection in murine neuronal cells leads to neurotoxicity and death and that TNF-α release plays an important role in this process [42], suggesting that inflammatory mechanisms triggered by ZIKV infection may differ across different experimental conditions. Although the death receptors pathway may not be the main trigger of cell death in our experimental conditions, we do not rule out that ZIKV infection triggers the production of proinflammatory cytokines in SH-SY5Y cells and that these may play a role in the process. Accordingly, previous data from our group showed that ZIKV infection in SH-SY5Y cells induces the production of several proinflammatory molecules including the proapoptotic chemokine CXCL10 [43], suggesting that indeed, ZIKV infection triggers an intricate inflammation process that needs to be further explored. 

To the best of our knowledge, this is the first study that evaluates the autocrine role of ROS in ZIKV-induced death in human neuronal-like cells in vitro. Our data excluded excessive ROS production as a key process in ZIKV-induced death of SH-SY5Y cells. Interestingly, a previous report in human glioblastoma and human liver carcinoma cell lines showed that ZIKV induces oxidative stress with the downregulation of antioxidant enzymes [44], and another previous study about in vitro ZIKV infection in human astrocytes showed that it leads to oxidative stress that can be reversed by ascorbic acid (antioxidant) treatment [15]. Altogether, these results suggest that the oxidative stress response pathways triggered by ZIKV may be different across different cell types and needs further assessment in order to identify the particularities of each experimental setting. In fact, this corroborates the notion that each cell displays a unique redox environment and is highly dependent on the metabolism of cells and compartmentation of redox regulation components [45,46]. In this sense, one possibility is that ZIKV induces antioxidant compensatory responses that inhibit ROS detrimental effects. A previous study on WNV infection showed that ROS induction is followed by an antioxidant compensatory response that is sufficient to also overcome mitochondrial damage induced by both viral infection and Arsenite treatment (a commonly used cellular stressor) [47]. Moreover, another previous report showed that Usutu virus (USUV), a flavivirus closely related to ZIKV and DENV, showed antioxidant capacity in both murine neuroblastoma (Neuro2a) and Vero cell lines [48]. Whether ZIKV triggers a similar antioxidant mechanism to avoid oxidative stress in SH-SY5Y cells still remains to be determined.

In conclusion, our study highlights some essential aspects of the apoptotic pathway triggered by ZIKV infection in SH-SY5Y cells, and our results indicate that excessive ROS production is not the main trigger of SH-SY5Y cells death in ZIKV infection, providing a better comprehension of the apoptotic mechanism triggered by ZIKV in neuronal-like cells.

## Data Availability

Gene expression data are deposited on the Gene Expression Omnibus online repository under the accession number GSE162096.

## Supplementary Materials

The following are available online at https://www.mdpi.com/article/10.3390/v13112111/s1. Figure S1: ZIKV-induced apoptosis is partially dependent on caspase activation (related to Figure 2). (a) Mock- and ZIKV-infected (MOI 0.5) SH-SY5Y cell supernatant were harvested at 2 dpi, and viral titer was analyzed by plaque assay using 10-fold serial dilution of mock and infected samples. (b) Mock- and ZIKV-infected (MOI 5) SH-SY5Y cells were treated with Q-VD-OPh (10 µM), and cell viability was assessed by MTS reduction assay at 5 dpi. Data represented as mean ± SD of one experiment performed in triplicate. Statistical analysis performed by one-way ANOVA followed by Tukey’s post-test, ** *p* <0.01, n.s.—not-significant, according to Tukey’s post-test. (c) qRT-PCR analysis of viral supernatant titers of mock- and ZIKV-infected (MOI 5) cells untreated or treated with 25 μM of Q-VD-Oph (Q-VD-Oph 25), or with vehicle (DMSO), and harvested at 3 and 4 dpi. Representative data of one experiment performed in triplicate, Figure S2: Expression profile of genes involved in apoptosis and survival response pathways in ZIKV-infected SH-SY5Y cells (related to Figure 3). Mock- and ZIKV-infected (MOI 5) SH-SY5Y cells were harvested and processed at 1 and 2 dpi for gene expression analysis. Expression levels of genes related to (a) antiapoptotic and (b) survival responses, (c) death receptors, (d) death receptors ligands, (e) death receptors pathway downstream kinases, and (f) death receptors adaptor proteins. Data represented as mean ± SD of one experiment performed in duplicate. N.D.—expression not detected. Statistical analysis performed by one-way ANOVA followed by Tukey’s post-test. ns—non-significant, * *p* < 0.05, ** *p* < 0.01, *** *p* < 0.001, **** *p* < 0.0001, according to Tukey’s post-test, Figure S3: ZIKV infection triggers intracellular redistribution of mitochondrial marker Mitotracker DeepRed FM. Uninfected (upper panels) and ZIKV-infected (MOI 5, lower panels) SH-SY5Y cells were harvested 2 dpi and processed for fluorescence microscopy. Cells were stained with Mitotracker DeepRed FM mitochondrial probe (red) and labeled with anti-flavivirus E (4G2 primary antibody) and AlexaFluor-488 anti-mouse IgG (green). Scale bar: 20 μM. Insets magnification:2X, Figure S4: ZIKV-induced apoptosis does not require TNF-α synthesis. (a–d) Mock- and ZIKV-infected (MOI 5) SH-SY5Y cells were treated with Brefeldin A for 1 h prior to antibody labeling and then processed for intracellular cytokine labeling and FCM analysis. Representative data of two independent experiments performed in duplicate. (a) Histograms showing mean fluorescence intensity (MFI) of unstained cells (gray), mock- (blue), and ZIKV-infected cells (red) of target ligands as indicated. Intracellular time-course detection of (b) TNF-α, (c) TWEAK, and (d) FasL performed at the indicated times post-infection. Data represented as mean ± SD of one experiment performed in duplicate. ns—not significant compared to mock-infected cells. Statistical analysis was performed by one-way ANOVA followed by Tukey’s post-test. (e,f) Mock- and ZIKV-infected (MOI 5) SH-SY5Y cells were treated with pentoxifylline (PTX) at 1 mM and 2 mM, harvested at 4 dpi, stained with LIVE/DEAD Viability/Cytotoxicity Kit, and analyzed by FCM. (e) Cell death percentage within the population of mock- and ZIKV-infected cells. Values represent the frequency of live cells (calcein AM high/EtHD1-) and dead cells (calcein AM low/EtHD1+). (f) Frequency (% parent) of live cells in mock- and ZIKV-infected samples untreated or treated with PTX. Data represented as mean ± SD of one experiment performed in quadruplicate. ns—not-significant. Statistical analysis was performed by one-Way ANOVA followed by Tukey’s post-test, Figure S5: Mock- and ZIKV-infected (MOI 5) SH-SY5Y cells treated with NAC (0.5 mM, 0.75 mM, and 1 mM) were harvested at the indicated timepoints post-infection, stained with Annexin-VI/7-AAD, and analyzed by FCM. (a) Frequency (percentage parent) of double-positive cells (Annexin-V+/7-AAD+). Data are represented as mean ± SD of one experiment performed in triplicate. (b) Cell death kinetics of mock- or NAC-treated SH-SY5Y cells during ZIKV infection. Pre—treatment before viral inoculation; post—treatment after viral inoculation, Table S1: Expression levels of 42 genes related to survival and death responses, Table S2: Experimental details relative to supernatant samples of MOCK- and ZIKV-infected cells analyzed for TNF-α detection by ELISA immunoassay.

## References

[1] Pierson T.C., Diamond M.S. (2018). The Emergence of Zika Virus and Its New Clinical Syndromes. Nature.

[2] Liu J., Li Q., Li X., Qiu Z., Li A., Liang W., Chen H., Cai X., Chen X., Duan X. (2018). Zika Virus Envelope Protein Induces G2/M Cell Cycle Arrest and Apoptosis via an Intrinsic Cell Death Signaling Pathway in Neuroendocrine PC12 Cells. Int. J. Biol. Sci..

[3] Gabriel E., Ramani A., Karow U., Gottardo M., Natarajan K., Gooi L.M., Goranci-Buzhala G., Krut O., Peters F., Nikolic M. (2017). Recent Zika Virus Isolates Induce Premature Differentiation of Neural Progenitors in Human Brain Organoids. Cell Stem Cell.

[4] Rosa-Fernandes L., Cugola F.R., Russo F.B., Kawahara R., de Melo Freire C.C., Leite P.E.C., Bassi Stern A.C., Angeli C.B., de Oliveira D.B.L., Melo S.R. (2019). Zika Virus Impairs Neurogenesis and Synaptogenesis Pathways in Human Neural Stem Cells and Neurons. Front. Cell. Neurosci..

[5] Russo F.B., Jungmann P., Beltrao-Braga P.C.B. (2017). Zika Infection and the Development of Neurological Defects. Cell. Microbiol..

[6] D’Arcy M.S. (2019). Cell Death: A Review of the Major Forms of Apoptosis, Necrosis and Autophagy. Cell Biol. Int..

[7] Elmore S. (2007). Apoptosis: A Review of Programmed Cell Death. Toxicol. Pathol..

[8] Galluzzi L., Vitale I., Abrams J.M., Alnemri E.S., Baehrecke E.H., Blagosklonny M.V., Dawson T.M., Dawson V.L., El-Deiry W.S., Fulda S. (2012). Molecular Definitions of Cell Death Subroutines: Recommendations of the Nomenclature Committee on Cell Death 2012. Cell Death Differ..

[9] Bras M., Queenan B., Susin S.A. (2005). Programmed Cell Death via Mitochondria: Different Modes of Dying. Biochem. Biokhimiia.

[10] Han X., Wang J., Yang Y., Qu S., Wan F., Zhang Z., Wang R., Li G., Cong H. (2021). Zika Virus Infection Induced Apoptosis by Modulating the Recruitment and Activation of Proapoptotic Protein Bax. J. Virol..

[11] Di Meo S., Reed T.T., Venditti P., Victor V.M. (2016). Role of ROS and RNS Sources in Physiological and Pathological Conditions. Oxidative Med. Cell. Longev..

[12] Srivastava R., Kalita J., Khan M.Y., Misra U.K. (2009). Free Radical Generation by Neurons in Rat Model of Japanese Encephalitis. Neurochem. Res..

[13] Olagnier D., Peri S., Steel C., van Montfoort N., Chiang C., Beljanski V., Slifker M., He Z., Nichols C.N., Lin R. (2014). Cellular Oxidative Stress Response Controls the Antiviral and Apoptotic Programs in Dengue Virus-Infected Dendritic Cells. PLoS Pathog..

[14] Diteepeng T., Khongwichit S., Paemanee A., Roytrakul S., Smith D.R. (2019). Proteomic Analysis of Monkey Kidney LLC-MK2 Cells Infected with a Thai Strain Zika Virus. Arch. Virol..

[15] Ledur P.F., Karmirian K., Pedrosa C.S.G., Souza L.R.Q., Assis-de-Lemos G., Martins T.M., Ferreira J.D.C.C.G., Reis G.F.D.A., Silva E.S., Silva D. (2020). Zika Virus Infection Leads to Mitochondrial Failure, Oxidative Stress and DNA Damage in Human IPSC-Derived Astrocytes. Sci. Rep..

[16] Kovalevich J., Langford D., Amini S., White M.K. (2013). Considerations for the Use of SH-SY5Y Neuroblastoma Cells in Neurobiology. Neuronal Cell Culture: Methods and Protocols.

[17] Donald C.L., Brennan B., Cumberworth S.L., Rezelj V.V., Clark J.J., Cordeiro M.T., Freitas de Oliveira França R., Pena L.J., Wilkie G.S., Da Silva Filipe A. (2016). Full Genome Sequence and SfRNA Interferon Antagonist Activity of Zika Virus from Recife, Brazil. PLoS Negl. Trop. Dis..

[18] Lanciotti R.S., Kosoy O.L., Laven J.J., Velez J.O., Lambert A.J., Johnson A.J., Stanfield S.M., Duffy M.R. (2007). Genetic and Serologic Properties of Zika Virus Associated with an Epidemic, Yap State, Micronesia. Emerg. Infect. Dis..

[19] Ferraris P., Cochet M., Hamel R., Gladwyn-Ng I., Alfano C., Diop F., Garcia D., Talignani L., Montero-Menei C.N., Nougairède A. (2019). Zika Virus Differentially Infects Human Neural Progenitor Cells According to Their State of Differentiation and Dysregulates Neurogenesis through the Notch Pathway. Emerg. Microbes Infect..

[20] Li C., Xu D., Ye Q., Hong S., Jiang Y., Liu X., Zhang N., Shi L., Qin C.-F., Xu Z. (2016). Zika Virus Disrupts Neural Progenitor Development and Leads to Microcephaly in Mice. Cell Stem Cell.

[21] Tang H., Hammack C., Ogden S.C., Wen Z., Qian X., Li Y., Yao B., Shin J., Zhang F., Lee E.M. (2016). Zika Virus Infects Human Cortical Neural Progenitors and Attenuates Their Growth. Cell Stem Cell.

[22] Deree J., Martins J.O., Melbostad H., Loomis W.H., Coimbra R. (2008). Insights into the Regulation of TNF-Alpha Production in Human Mononuclear Cells: The Effects of Non-Specific Phosphodiesterase Inhibition. Clinics.

[23] Muchhala S.K., Benzeroual K.E. (2012). Pentoxifylline Suppressed LPS-Induced Inflammatory and Apoptotic Signaling in Neuronal Cells. Adv. Biosci. Biotechnol..

[24] Gupta K.J., Igamberdiev A.U. (2016). Reactive Nitrogen Species in Mitochondria and Their Implications in Plant Energy Status and Hypoxic Stress Tolerance. Front. Plant Sci..

[25] Newsholme P., Haber E.P., Hirabara S.M., Rebelato E.L.O., Procopio J., Morgan D., Oliveira-Emilio H.C., Carpinelli A., Curi R. (2007). Diabetes Associated Cell Stress and Dysfunction: Role of Mitochondrial and Non-Mitochondrial ROS Production and Activity. J. Physiol..

[26] Hastings A.K., Hastings K., Uraki R., Hwang J., Gaitsch H., Dhaliwal K., Williamson E., Fikrig E. (2019). Loss of the TAM Receptor Axl Ameliorates Severe Zika Virus Pathogenesis and Reduces Apoptosis in Microglia. iScience.

[27] Monel B., Compton A.A., Bruel T., Amraoui S., Burlaud-Gaillard J., Roy N., Guivel-Benhassine F., Porrot F., Génin P., Meertens L. (2017). Zika Virus Induces Massive Cytoplasmic Vacuolization and Paraptosis-like Death in Infected Cells. EMBO J..

[28] Souza B.S.F., Sampaio G.L.A., Pereira C.S., Campos G.S., Sardi S.I., Freitas L.A.R., Figueira C.P., Paredes B.D., Nonaka C.K.V., Azevedo C.M. (2016). Zika Virus Infection Induces Mitosis Abnormalities and Apoptotic Cell Death of Human Neural Progenitor Cells. Sci. Rep..

[29] Zhang F., Hammack C., Ogden S.C., Cheng Y., Lee E.M., Wen Z., Qian X., Nguyen H.N., Li Y., Yao B. (2016). Molecular Signatures Associated with ZIKV Exposure in Human Cortical Neural Progenitors. Nucleic Acids Res..

[30] Chatel-Chaix L., Cortese M., Romero-Brey I., Bender S., Neufeldt C.J., Fischl W., Scaturro P., Schieber N., Schwab Y., Fischer B. (2016). Dengue Virus Perturbs Mitochondrial Morphodynamics to Dampen Innate Immune Responses. Cell Host Microbe.

[31] Waterhouse N.J., Goldstein J.C., von Ahsen O., Schuler M., Newmeyer D.D., Green D.R. (2001). Cytochrome c Maintains Mitochondrial Transmembrane Potential and ATP Generation after Outer Mitochondrial Membrane Permeabilization during the Apoptotic Process. J. Cell Biol..

[32] Yang T.-C., Shiu S.-L., Chuang P.-H., Lin Y.-J., Wan L., Lan Y.-C., Lin C.-W. (2009). Japanese Encephalitis Virus NS2B-NS3 Protease Induces Caspase 3 Activation and Mitochondria-Mediated Apoptosis in Human Medulloblastoma Cells. Virus Res..

[33] Jan J.T., Chen B.H., Ma S.H., Liu C.I., Tsai H.P., Wu H.C., Jiang S.-Y., Yang K.-D., Shaio M.-F. (2000). Potential Dengue Virus-Triggered Apoptotic Pathway in Human Neuroblastoma Cells: Arachidonic Acid, Superoxide Anion, and NF-KappaB Are Sequentially Involved. J. Virol..

[34] Kleinschmidt M.C., Michaelis M., Ogbomo H., Doerr H.-W., Cinatl J. (2007). Inhibition of Apoptosis Prevents West Nile Virus Induced Cell Death. BMC Microbiol..

[35] Bell J.H., Herrera A.H., Li Y., Walcheck B. (2007). Role of ADAM17 in the Ectodomain Shedding of TNF- and Its Receptors by Neutrophils and Macrophages. J. Leukoc. Biol..

[36] Clark A. (2000). Post-Transcriptional Regulation of pro-Inflammatory Gene Expression. Arthritis Res..

[37] Anderson P. (2000). Post-Transcriptional Regulation of Tumour Necrosis Factor α Production. Ann. Rheum. Dis..

[38] Stamou P., Kontoyiannis D.L. (2010). Posttranscriptional Regulation of TNF MRNA: A Paradigm of Signal-Dependent MRNA Utilization and Its Relevance to Pathology. Current Directions in Autoimmunity.

[39] Crawford E.K., Ensor J.E., Kalvakolanu I., Hasday J.D. (1997). The Role of 3′ Poly(A) Tail Metabolism in Tumor Necrosis Factor-α Regulation. J. Biol. Chem..

[40] Hanners N.W., Eitson J.L., Usui N., Richardson R.B., Wexler E.M., Konopka G., Schoggins J.W. (2016). Western Zika Virus in Human Fetal Neural Progenitors Persists Long Term with Partial Cytopathic and Limited Immunogenic Effects. Cell Rep..

[41] Stefanik M., Formanova P., Bily T., Vancova M., Eyer L., Palus M., Salat J., Braconi C.T., Zanotto P.M.d.A., Gould E.A. (2018). Characterisation of Zika Virus Infection in Primary Human Astrocytes. BMC Neurosci..

[42] Olmo I.G., Carvalho T.G., Costa V.V., Alves-Silva J., Ferrari C.Z., Izidoro-Toledo T.C., da Silva J.F., Teixeira A.L., Souza D.G., Marques J.T. (2017). Zika Virus Promotes Neuronal Cell Death in a Non-Cell Autonomous Manner by Triggering the Release of Neurotoxic Factors. Front. Immunol..

[43] Lima M.C., de Mendonca L.R., Rezende A.M., Carrera R.M., Anibal-Silva C.E., Demers M., D’Aiuto L., Wood J., Chowdari K.V., Griffiths M. (2019). The Transcriptional and Protein Profile from Human Infected Neuroprogenitor Cells Is Strongly Correlated to Zika Virus Microcephaly Cytokines Phenotype Evidencing a Persistent Inflammation in the CNS. Front. Immunol..

[44] Almeida L.T., Ferraz A.C., da Silva Caetano C.C., da Silva Menegatto M.B., dos Santos Pereira Andrade A.C., Lima R.L.S., Camini F.C., Pereira S.H., da Silva Pereira K.Y., de Mello Silva B. (2020). Zika Virus Induces Oxidative Stress and Decreases Antioxidant Enzyme Activities in Vitro and in Vivo. Virus Res..

[45] Circu M.L., Aw T.Y. (2008). Glutathione and Apoptosis. Free Radic. Res..

[46] Milkovic L., Cipak Gasparovic A., Cindric M., Mouthuy P.A., Zarkovic N. (2019). Short Overview of ROS as Cell Function Regulators and Their Implications in Therapy Concepts. Cells.

[47] Basu M., Courtney S.C., Brinton M.A. (2017). Arsenite-Induced Stress Granule Formation Is Inhibited by Elevated Levels of Reduced Glutathione in West Nile Virus-Infected Cells. PLoS Pathog..

[48] Blázquez A.B., Martín-Acebes M.A., Poderoso T., Saiz J.C. (2021). Relevance of Oxidative Stress in Inhibition of Eif2 Alpha Phosphorylation and Stress Granules Formation during Usutu Virus Infection. PLoS Negl. Trop. Dis..

