# Supplementary material for: Reactive Oxygen Species (ROS) Are Not a Key Determinant for Zika Virus-Induced Apoptosis in SH-SY5Y Neuroblastoma Cells
Source: Viruses. 2021 Oct 20;13(11):2111. doi: 10.3390/v13112111 (PMC8622630; doi:10.3390/v13112111)
Supplement: Supplementary file 1 [file viruses-13-02111-s001.zip › viruses-1321845-supplementary.pdf]

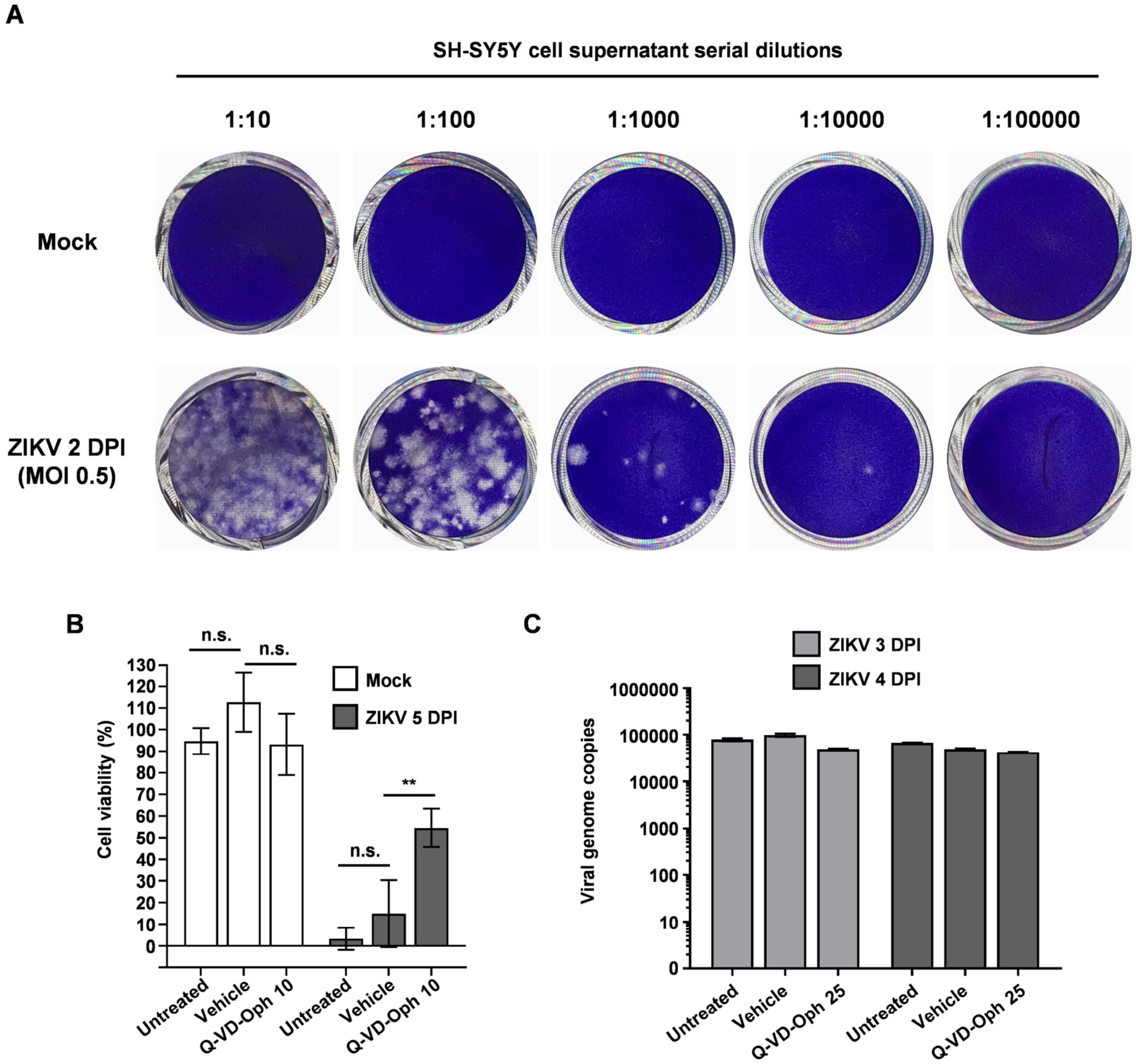

Figure S1: ZIKV-induced apoptosis is partially dependent on caspase activation (related to Figure 2). (a) Mock- and ZIKV-infected (MOI 0.5) SH-SY5Y cell supernatant were harvested at 2 dpi, and viral titer was analyzed by plaque assay using 10-fold serial dilution of mock and infected samples. (b) Mock- and ZIKV-infected (MOI 5) SH-SY5Y cells were treated with Q-VD-OPh (10 μM), and cell viability was assessed by MTS reduction assay at 5 dpi. Data represented as mean ± SD of one experiment performed in triplicate. Statistical analysis performed by one-way ANOVA followed by Tukey's post-test, ** p <0.01, n.s.—not-significant, according to Tukey's post-test. (c) qRT-PCR analysis of viral supernatant titers of mock- and ZIKV-infected (MOI 5) cells untreated or treated with 25 μM of Q-VD-Oph (Q-VD-Oph 25), or with vehicle (DMSO), and harvested at 3 and 4 dpi. Representative data of one experiment performed in triplicate

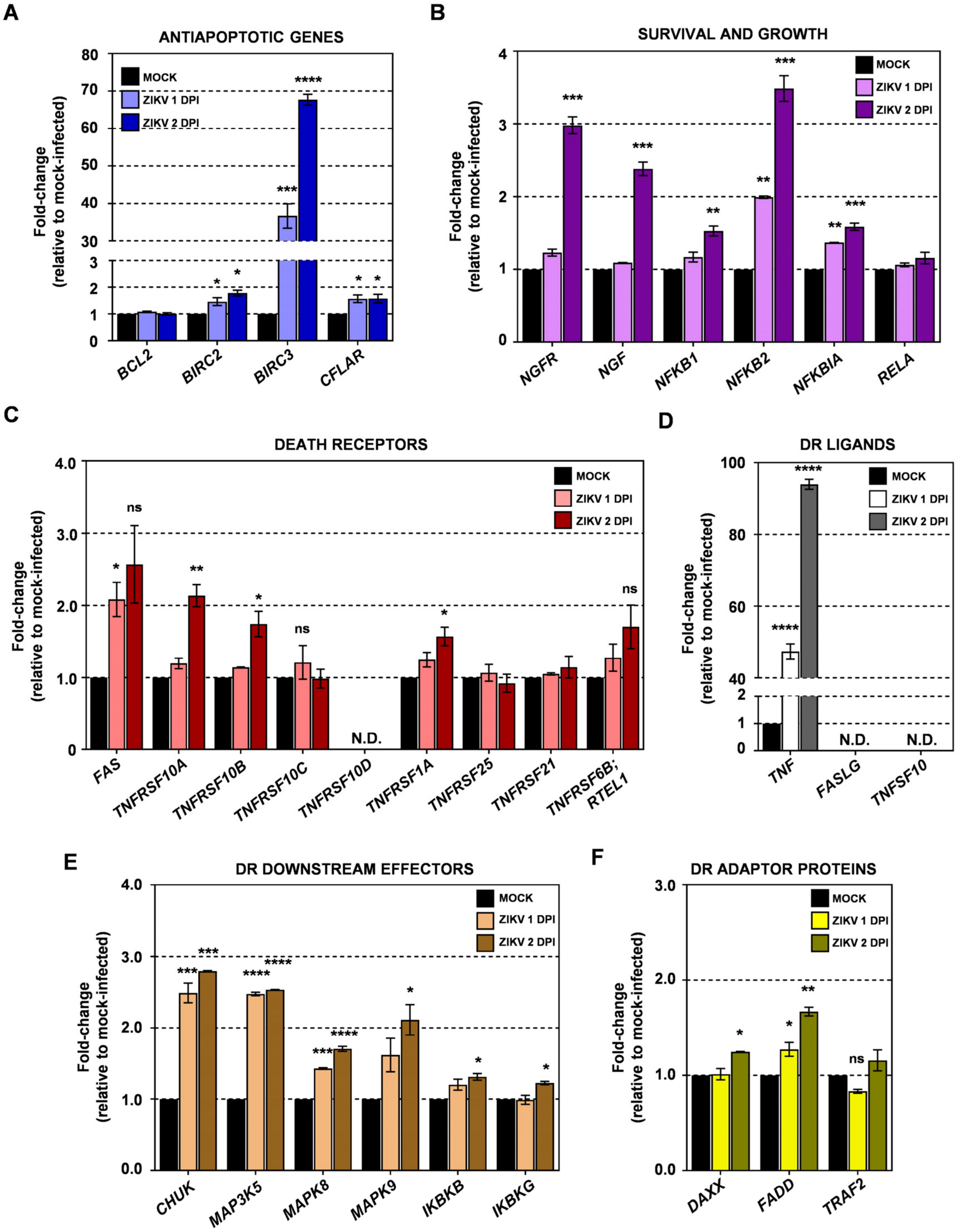

Figure S2: Expression profile of genes involved in apoptosis and survival response pathways in ZIKV-infected SH-SY5Y cells (related to Figure 3). Mock- and ZIKV-infected (MOI 5) SH-SY5Y cells were harvested and processed at 1 and 2 dpi for gene expression analysis. Expression levels of genes related to (a) antiapoptotic and (b) survival responses, (c) death receptors, (d) death receptors ligands, (e) death receptors pathway downstream kinases, and (f) death receptors adaptor proteins. Data represented as mean ± SD of one experiment performed in duplicate. N.D.—expression not detected. Statistical analysis performed by one-way ANOVA followed by Tukey's post-test. ns—non-significant, * $p < 0.05$, ** $p < 0.01$, *** $p < 0.001$, **** $p < 0.0001$, according to Tukey's post-test

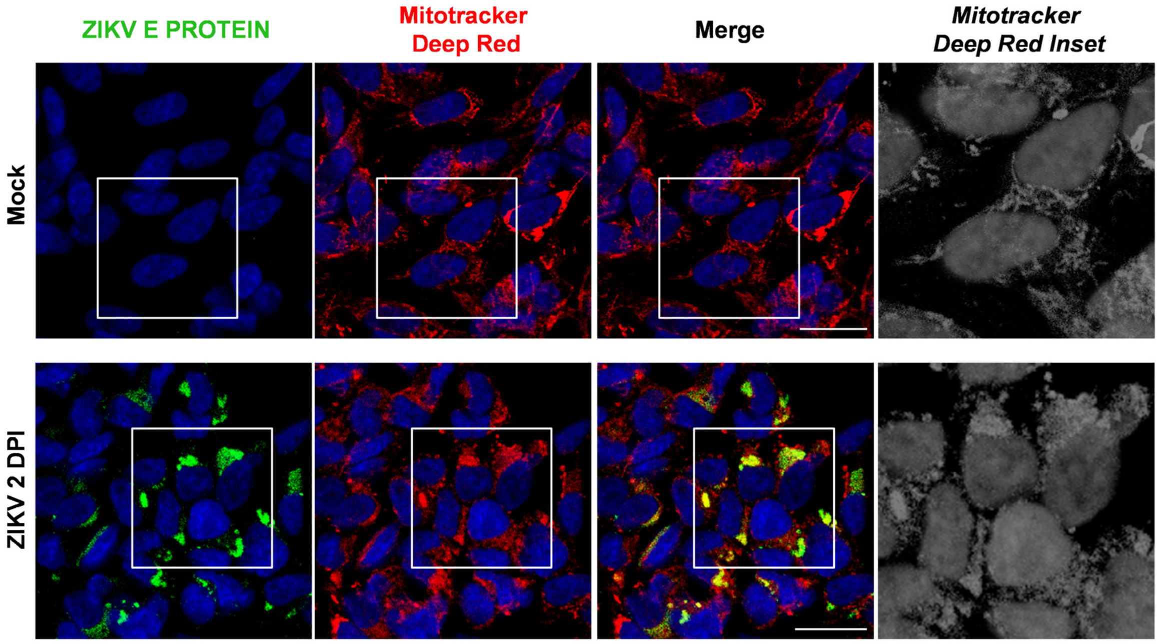

Figure S3: ZIKV infection triggers intracellular redistribution of mitochondrial marker Mitotracker DeepRed FM. Uninfected (upper panels) and ZIKV-infected (MOI 5, lower panels) SH-SY5Y cells were harvested 2 dpi and processed for fluorescence microscopy. Cells were stained with Mitotracker DeepRed FM mitochondrial probe (red) and labeled with anti-flavivirus E (4G2 primary antibody) and AlexaFluor-488 anti-mouse IgG (green). Scale bar: 20 µM. Insets magnification:2X

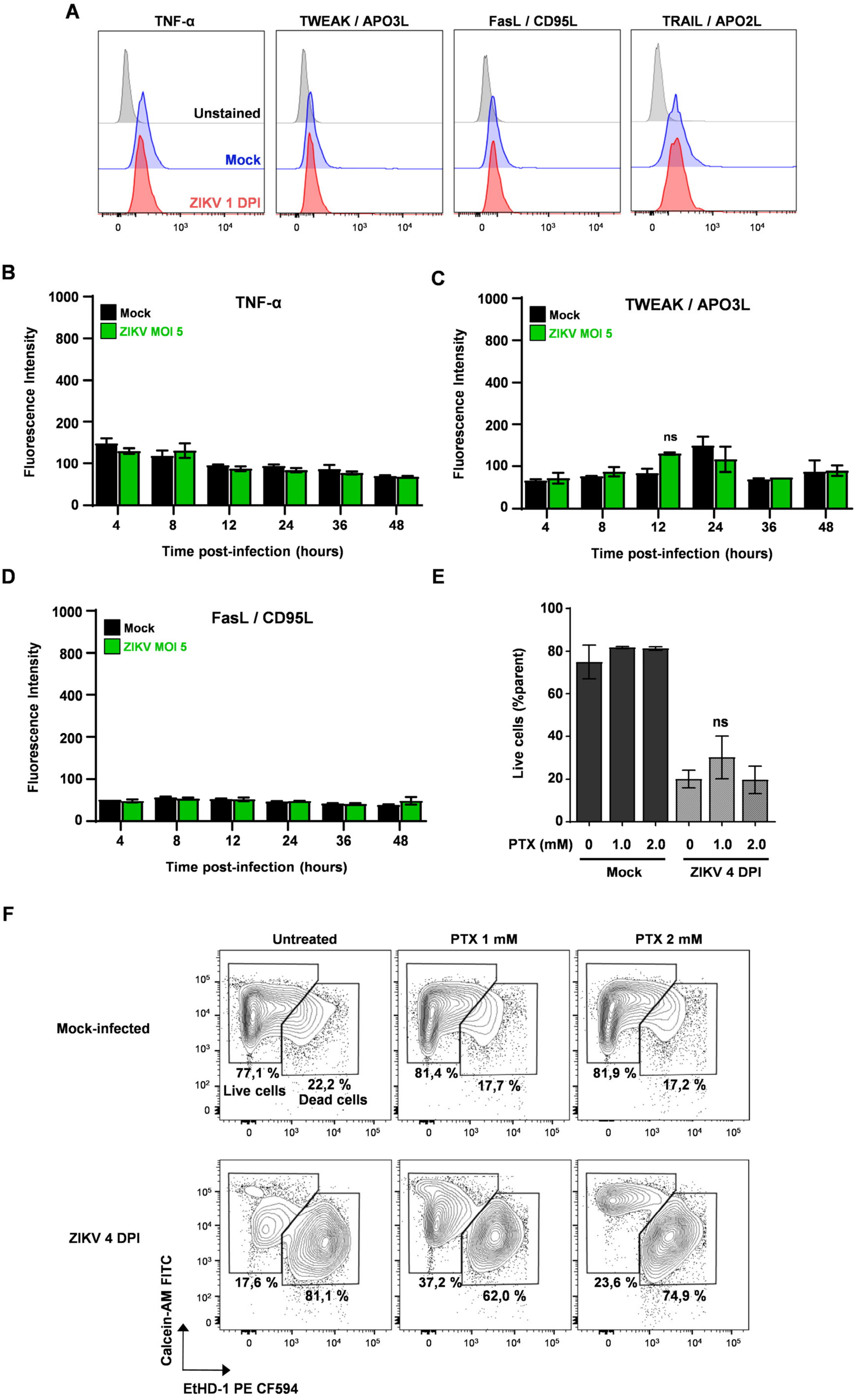
A
TNF-α
TWEAK / APO3L
FasL / CD95L
TRAIL / APO2L
Unstained
Mock
ZIKV 1 DPI
B
TNF-α
Mock
ZIKV MOI 5
Fluorescence Intensity
Time post-infection (hours)
C
TWEAK / APO3L
ns
D
FasL / CD95L
E
Live cells (%parent)
ns
PTX (mM)
Mock
ZIKV 4 DPI
F
Untreated
PTX 1 mM
PTX 2 mM
Mock-infected
77,1 %
Live cells
22,2 %
Dead cells
81,4 %
17,7 %
81,9 %
17,2 %
ZIKV 4 DPI
17,6 %
81,1 %
37,2 %
62,0 %
23,6 %
74,9 %
Calcein-AM FITC
EtHD-1 PE CF594

Figure S4: ZIKV-induced apoptosis does not require TNF-α synthesis. (a-d) Mock- and ZIKV-infected (MOI 5) SH-SY5Y cells were treated with Brefeldin A for 1 h prior to antibody labeling and then processed for intracellular cytokine labeling and FCM analysis. Representative data of two independent experiments performed in duplicate. (a) Histograms showing mean fluorescence intensity (MFI) of unstained cells (gray), mock- (blue), and ZIKV-infected cells (red) of target ligands as indicated. Intracellular time-course detection of (b) TNF-α, (c) TWEAK, and (d) FasL performed at the indicated times post-infection. Data represented as mean ± SD of one experiment performed in duplicate. ns—not significant compared to mock-infected cells. Statistical analysis was performed by one-way ANOVA followed by Tukey's post-test. (e-f) Mock- and ZIKV-infected (MOI 5) SH-SY5Y cells were treated with pentoxifylline (PTX) at 1 mM and 2 mM, harvested at 4 dpi, stained with LIVE/DEAD Viability/Cytotoxicity Kit, and analyzed by FCM. (e) Cell death percentage within the population of mock- and ZIKV-infected cells. Values represent the frequency of live cells (calcein AM high/EtHD1-) and dead cells (calcein AM low/EtHD1+). (f) Frequency (% parent) of live cells in mock- and ZIKV-infected samples untreated or treated with PTX. Data represented as mean ± SD of one experiment performed in quadruplicate. ns—not-significant. Statistical analysis was performed by one-Way ANOVA followed by Tukey's post-test

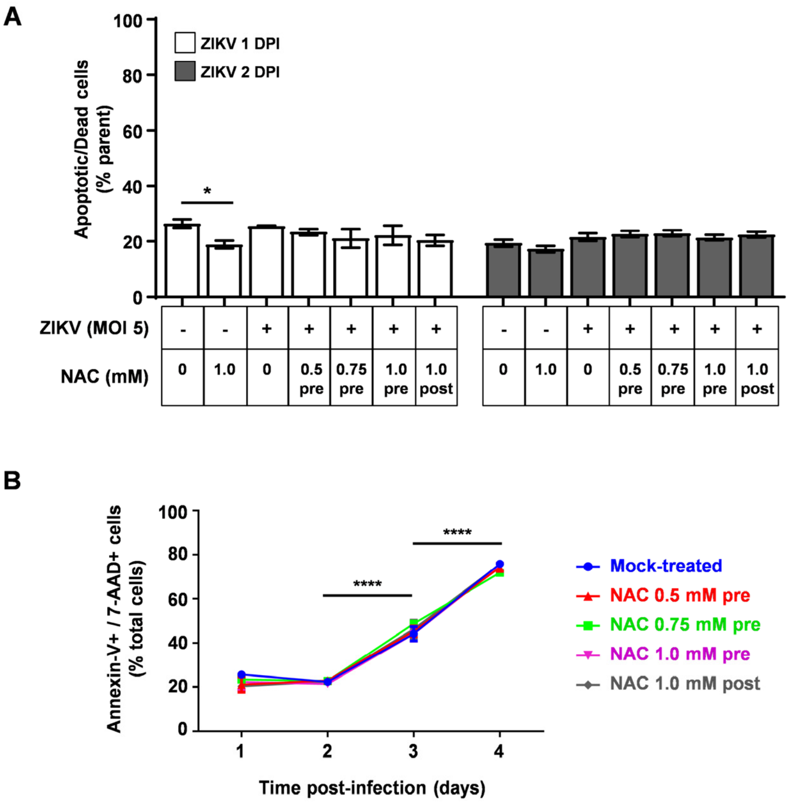

Figure S5: Mock- and ZIKV-infected (MOI 5) SH-SY5Y cells treated with NAC (0.5 mM, 0.75 mM, and 1 mM) were harvested at the indicated timepoints post-infection, stained with Annexin-VI/7-AAD, and analyzed by FCM. (a) Frequency (percentage parent) of double-positive cells (Annexin-V+/7-AAD+). Data are represented as mean ± SD of one experiment performed in triplicate. (b) Cell death kinetics of mock- or NAC-treated SH-SY5Y cells during ZIKV infection. Pre—treatment before viral inoculation; post—treatment after viral inoculation
